# The Functions of Metallothionein and ZIP and ZnT Transporters: An Overview and Perspective

**DOI:** 10.3390/ijms17030336

**Published:** 2016-03-04

**Authors:** Tomoki Kimura, Taiho Kambe

**Affiliations:** 1 Department of Life Science, Faculty of Science and Engineering, Setsunan University, Neyagawa, Osaka 572-8508, Japan; 2 Division of Integrated Life Science, Graduate School of Biostudies, Kyoto University, Kyoto 606-8502, Japan

**Keywords:** zinc, metallothionein, ZIP and ZnT transporter, chaperone

## Abstract

Around 3000 proteins are thought to bind zinc *in vivo*, which corresponds to ~10% of the human proteome. Zinc plays a pivotal role as a structural, catalytic, and signaling component that functions in numerous physiological processes. It is more widely used as a structural element in proteins than any other transition metal ion, is a catalytic component of many enzymes, and acts as a cellular signaling mediator. Thus, it is expected that zinc metabolism and homeostasis have sophisticated regulation, and elucidating the underlying molecular basis of this is essential to understanding zinc functions in cellular physiology and pathogenesis. In recent decades, an increasing amount of evidence has uncovered critical roles of a number of proteins in zinc metabolism and homeostasis through influxing, chelating, sequestrating, coordinating, releasing, and effluxing zinc. Metallothioneins (MT) and Zrt- and Irt-like proteins (ZIP) and Zn transporters (ZnT) are the proteins primarily involved in these processes, and their malfunction has been implicated in a number of inherited diseases such as acrodermatitis enteropathica. The present review updates our current understanding of the biological functions of MTs and ZIP and ZnT transporters from several new perspectives.

## 1. Introduction

**Figure 1:**
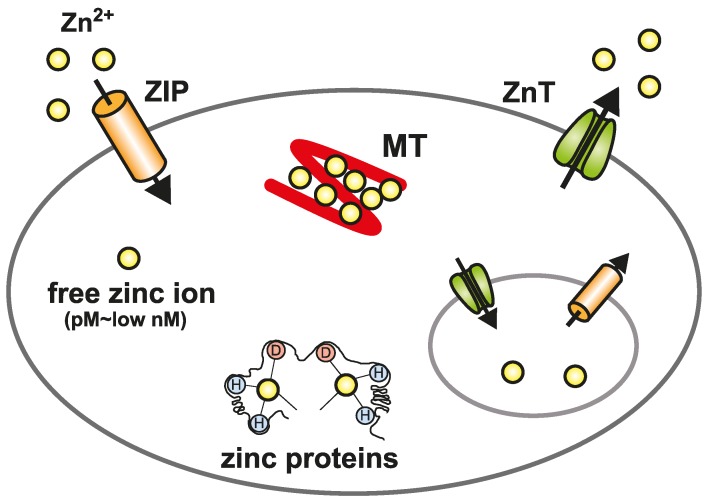
Cellular zinc homeostasis is controlled by the cooperative function of metallothioneins (MT) and Zrt- and Irt-like proteins (ZIP) and Zn transporters (ZnT). The mobilization of zinc into or out of the cytosol is directed by two zinc transporter families, ZIP and ZnT. In the cytosol, MTs bind zinc to reserve, buffer, and chelate. Zinc is compartmentalized into or out of intracellular organelles and vesicles by ZnT and ZIP transporters. Because of the binding of zinc to many different proteins, the free zinc ion concentration in the cytosol is estimated to be well below pM–low nM levels.

Following the uptake of zinc by cells, it is distributed within the cytoplasm (50%), nucleus (30%–40%), and cell membrane (10%) [1,2]. Cellular zinc is then available as four pools [2,3]. First, it can bind tightly to metalloproteins as a structural component or to metalloenzymes as a cofactor; Second, zinc binds metallothioneins (MTs) with a low affinity, which can occupy 5%–15% of the total cellular zinc pool [4]; Third, it can be compartmentalized into intracellular organelles and vesicles for zinc storage and as a supply for zinc-dependent proteins, which is mediated by zinc transporters [5,6]. As a result of the second and third functions, the fourth pool of cytosolic free zinc is maintained at a very low concentration (pM–low nM levels) [7,8,9]. MTs and two zinc transporter families, Zrt- and Irt-like proteins (ZIP, solute carrier 39A [SLC39A]) and Zn transporters (ZnT, SLC30A), play crucial roles to maintain this cellular zinc homeostasis [2,3,10,11,12] (Figure 1). In this review, we focus on recent progress to describe the physiological and biological functions of MTs and ZIP and ZnT transporters, and to provide a better understanding of zinc biology.

## 2. Physiological and Cellular Functional Properties of Metallothioneins (MTs)

### 2.1. MT Isoforms

**Figure 2:**
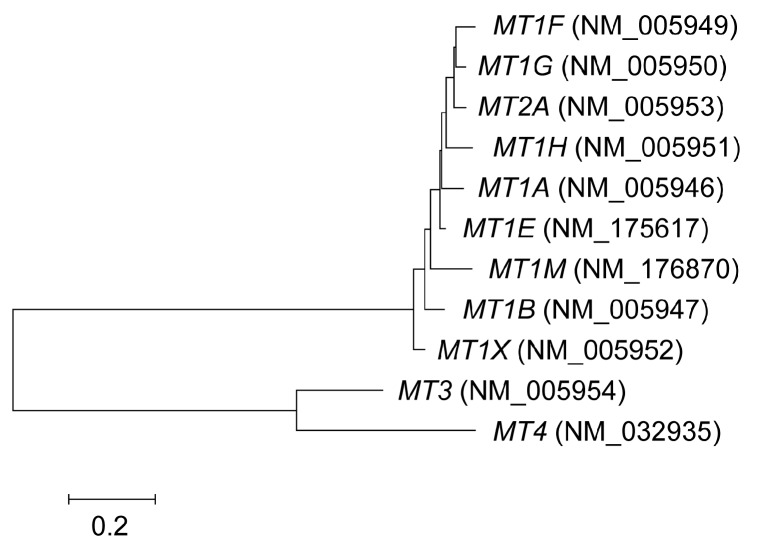
Phylogenetic tree of *MT* genes. The tree was constructed using coding sequences from NCBI RefSeq and the neighbor-joining method using MEGA6 software.

MTs are low-molecular-weight metal-binding proteins that lack disulfides and contain one-third cysteine residues. Human MTs have a total of 11 functional isoforms that can be divided into four classes, designated MT-1 to -4. These are encoded by eight active *MT1* genes (*MT1A*, *B*, *E*, *F*, *G*, *H*, *M*, and *X*), and a single copy of *MT2* (known as *MT2A*), *MT3*, and *MT*4. The human genome also contains five pseudo-*MT1* genes derived from duplication and loss-of-function mutations of the original parental *MT1*. However, it is not clear whether the eight active *MT1* genes have gained a new function. The mouse harbors single copies of *MT1*, *2*, *3*, and *4*. A tree topology study suggested that two rounds of duplication have occurred in the *MT* family [13] (Figure 2). The neighbor-joining method of analysis of human *MT* genes using MEGA6 software (http://www.megasoftware.net/) (Figure 2) suggested that the ancestor of *MT3*/*4* and *MT1/2* diverged, after which *MT3* and *MT4* seem to have separated. Conversely, the Bayesian and maximum likelihood methods of analysis [13] indicated that divergence of the *MT4* and *MT1/2/3* ancestor was the first step. In either case, following duplication of these genes, gain-of-function mutations are likely to have occurred in *MT1/2*, *MT3*, and *MT4*. MT-3 and -4 show a restricted cell type-specific expression pattern, with MT-3 being expressed mainly in the brain and MT-4 most abundant in certain epithelial tissues. These isoforms show specific roles in these tissues [14].

The ubiquitous MT isoforms MT-1 and -2 have been extensively investigated with regard to zinc metabolism. They are expressed in many cell types in various organs and tissues, as well as in most cultured cells, and their function is to maintain cellular zinc homeostasis and attenuate heavy metal-induced cytotoxicity by chelating these metals and lowering their intracellular concentrations. They also protect against several types of environmental stress through their radical scavenging properties [15].

It is unclear whether MT-1 and -2 have functional differences. Because of their similar amino acid sequences and inducibility in response to zinc and various stress conditions and compounds, most research studies of MT-1 and -2 have been done without separation. However, several lines of research show that the MT-1 and 2 isoforms have specific functions, which are summarized in Section 3.2. In the 1990s, two lines of *MT1/2* double knockout (KO) mice were established to examine the functional properties of MT-1 and -2 [16,17]. *MT1/2* KO mice were viable and reproduced normally when reared under standard laboratory conditions. The function of MT1/2 was shown not only in the protection against metals [16,17,18,19], oxidative stress [20], and carcinogens [21], but also in immune reactions and obesity (Table 1). *MT1/2* KO mice have a high sensitivity to lipopolysaccharide (LPS) and LPS/d-galactosamine, so represent an acute hepatic failure model [22,23]. However, the protective mechanisms are unclear. *MT1/2* KO mice also showed increased *Helicobacter pylori* (*H. pylori*)-induced gastric erosive lesions [24]. These lesions are associated with production of reactive oxygen species (ROS) from infiltrated macrophages and neutrophils. ROS scavenging activity of MT might be involved in the sensitization. A report detailing a decrease in interleukin (IL)-4 production in *MT1/2* KO mice, which is mediated by FcεRI-induced calcineurin (CaN)/nuclear factor of activated T-cell (NFAT) signaling pathway, suggests that the MT-dependent control of zinc homeostasis regulates IL-4 production in basophil granulocytes [25]. Interestingly, zinc transporter KO mice such as *Zip10* KO also showed an altered immune response [26,27]. We describe the KO phenotype of zinc transporters in Section 5.2 and Section 5.3.

As shown in Table 1, two strains of *MT1/2* KO mice exist. The first is 129SvCPJ [16], while the second was originally developed on a mixed genetic background of OLA129 and C57BL/6 strains [17] then backcrossed with C57BL/6J Jcl. The KO phenotype shown in Table 1 might be strain-specific. Other phenotypes, such as high-fat diet-induced obesity [28] and a shortened lifespan [29], appeared through unknown mechanisms.

Mutations in the Cu/Zn-superoxide dismutase (*SOD1*) gene cause one form of familial amyotrophic lateral sclerosis (ALS), a progressive disorder of motor neurons leading to death. *MT1/2* deficiency in mouse model of ALS involving mutated SOD1 (G93A SOD1) shows a reduction in survival compared with G93A SOD1 mice [30]. SOD1 is an enzyme that binds zinc, and abnormalities in this binding have been implicated in disease pathogenesis [31]. This study indicated that MT acts as a zinc chaperone for apo-SOD1. We discuss the possibility of MT being a zinc chaperone in more detail in Section 3.1 and Section 6.

### 2.2. The Zinc-Responsive Transcription Factor MTF-1

**Figure 3:**
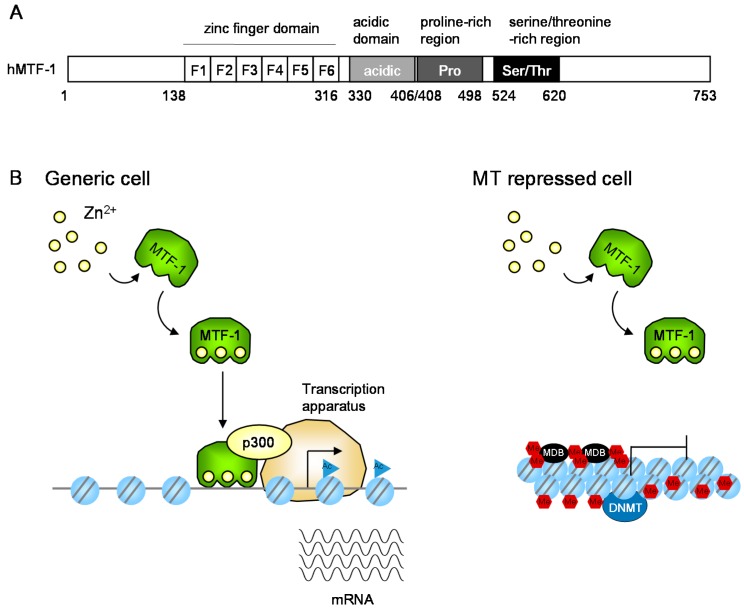
Expression regulation of *MT* gene expression. (**A**) Schematic representation of human metal response element-binding transcription factor-1 (MTF-1). The regions of the six-zinc fingers (F1–F6), acidic, proline-rich, and serine/threonine-rich domains are indicated by boxes and amino acid numbers; (**B**) Proposed molecular mechanisms in MT transcription in response to increases of intracellular free zinc. In generic cells, MTF-1 recruits the histone acetyltransferase p300 and increases MT transcription. In MT-repressed cells such as lymphosarcoma cells, and cancer cells, the promoter is highly methylated. DNA methyltransferase (DNMT) and methyl CpG binding proteins (MBD) are involved in the suppression. The epigenetic mechanism is described in Section 4.2. Ac, acetyl group; Me, methyl group; blue circle with two lines, nucleosome.

*MT1/2* transcription is regulated by the metal response element-binding transcription factor-1 (MTF-1), which is a zinc finger transcription factor that regulates metal-responsive gene expression [32] (Figure 3). In MTF-1 KO cells, *MT1/2* genes are silent. MTF-1 is an essential factor for basal and heavy metal-induced *MT1/2* expression. It possesses six Cys_2_His_2_ zinc fingers and three transcriptional activation domains, namely an acidic domain, a proline-rich region, and a serine/threonine-rich region. A simple metalloregulatory model suggests that MTF-1 has a low intrinsic binding affinity for zinc, and only binds zinc under zinc excess conditions. Specific zinc fingers in MTF-1 were shown to have zinc-binding affinities in the nM to sub-µM range [33], while canonical Cys_2_His_2_ zinc fingers typically bind zinc with higher affinity (10^−9^–10^−12^ M). Because zinc affinities of all six fingers are similar in MTF-1 (within ~10–50-fold of each other), zinc-sensing by MTF-1 is suggested to occur within a 100-fold or less range of accessible zinc concentration [34].

MTF-1 also regulates the zinc-responsive transcription of *ZnT1* and *ZnT2* [35,36] and represses the expression of *Zip10* [37,38], indicating that it plays an important role in zinc homeostasis. The tumor suppressor phosphatase and tensin homolog modulates the MTF-1-mediated expression of *ZnT1* and *MT* [39], suggesting a relationship between tumorigenesis and zinc homeostasis. Protein phosphatase 2A has also been reported to be involved in MT induction [40]. In addition to MTF-1, a recent finding revealed that another zinc finger transcription factor, ZNF658, is involved in the regulation of zinc transporter expression [41]. Thus, ZNF658 may cooperatively function as a transcriptional regulator with MTF-1 in the controlled cellular response to zinc availability.

## 3. Structural and Biochemical Functions of MTs

### 3.1. MT Functions in Physiological and Cellular Zinc Homeostasis

MT was first identified as a protein containing heavy metals such as cadmium and zinc [42]. MTs intracellularly bind these metals and lower their concentration at critical sites. A role for MT in cellular zinc homeostasis was predicted before being experimentally confirmed [4,14,43]. In one study, mouse fibroblasts were adapted to extreme zinc deprivation (<0.06 μM zinc *vs.* sub-μM levels in normal medium) by increasing *MT-1* mRNA expression through *MT1* amplification without MT protein accumulation. Apo-MT chelates zinc from the environment and increases intracellular zinc. When zinc levels were insufficient to stabilize the MT protein, the MT was rapidly proteolyzed. Zinc is then released by MT degradation, so the intracellular zinc concentration was kept constant. In contrast, when cells expressing *MT3* were deprived of zinc, cell proliferation was arrested and MT-3 protein levels persisted. MT1/2 were shown to scavenge extracellular zinc, not to compete with essential zinc-requiring proteins, and to be degraded, while MT3 was shown to compete for zinc and to exacerbate the zinc deficiency [44].

MTs can also function as a “zinc buffer” through their low-affinity binding, providing labile zinc for use by target proteins/enzymes when zinc is limited [45,46]. Specifically, several zinc-requiring apoenzymes can be reactivated by the transfer of zinc from zinc-saturated MT, which binds seven zinc ions. One study proposed the model that zinc-saturated Zn_7_-MT and Zn_6_-MT are the primary zinc-donating species for apo-carbonic anhydrase, a zinc-requiring enzyme [47]. In particular, MT is a source of zinc ions under conditions of redox signaling through the modifications of zinc-thiolate coordination environments [48,49], which contribute to the functions of zinc in cellular signaling [50]. It has also been reported that apo-MT removes zinc from the zinc finger transcription factors Sp1 and transcription factor IIIA *in vitro*, and eliminates their DNA-binding ability [51,52]. Thus, MT appears to act as a chaperone for zinc proteins/enzymes. This role involves the transfer of zinc from MT to proteins/enzymes via ligand exchange in zinc-mediated protein–protein interactions in the absence of freely released zinc ions, which is known as “the associative mechanism” [46,53,54] as seen in copper metabolism [55,56,57]. In this similar metabolism, several copper chaperones play critical roles in transferring cytosolic copper to target proteins/enzymes [55,56,57].

### 3.2. Specific Functions of MT-1 and -2 Isoforms

Although MT-1 and 2 have been largely studied together, the specific functions of these proteins have only begun to be elucidated [58,59]. For instance, comparing the stability of rat zinc-saturated MT-1 and MT-2, MT-2 was shown to degrade more slowly than MT-1 [58]. More recently, zinc-saturated MT-2 was found not to exist under normal physiological conditions [60], although it is unclear whether stability differs among zinc-unsaturated isoforms.

Several studies of *MT* single nucleotide polymorphisms (SNPs) showed that *MT2A* but not *MT1* SNPs are associated with an increased cancer risk [61,62,63] (Table 2). However, because the basal level of *MT2A* expression appears to be 5–10 times higher than that of *MT1X*, this association might be caused by *MT2A* expression levels. MT isoform-specific gene regulation mechanisms have also been reported. As an example, vascular endothelial growth factor was shown to induce human MT1G, but not MT2A, by regulating the E2F transcription factor [64], while bovine MT1A and 1E, but not MT2A, are induced through the Keap1-Nrf2 system [65]. Such isoform-specific functions may result from isoform-specific expression mechanisms.

However, MT isoform-specific linkage has also been reported. MT2A interacts with homeobox-containing 1 (HMBOX1) in human umbilical vascular endothelial cells to increase intracellular free zinc concentrations [66]. Knockdown of MT2A decreases intracellular free zinc, while treatment with a zinc chelator inhibited HMBOX1-regulated apoptosis and promoted HMBOX1-regulated autophagy. The interaction between MT1X, Akt, and tongue cancer resistance-associated protein 1 (TCRP1) in oral squamous cell carcinoma [67] was also reported. In this report, MT1X knockdown increased cisplatin-induced apoptosis. Although these MT isoforms might have specific functions, their amino acid sequences have a high level of homology. Therefore, further investigations are needed to clarify the mechanisms of isoform-specific function.

## 4. Novel Regulation of MT Expression

### 4.1. SNPs in the MT Promoter

As described in Section 2.2, *MT1/2* expression is regulated by the transcription factor MTF-1 (Figure 3). In *MTF-1* KO cells, *MT1/2* genes are silent, and therefore MTF-1 is essential for basal and heavy metal-induced *MT1/2* expression, while other transcription factors are also involved in the expression [71]. For example, signal transducer and activator of transcription 3 (STAT3) and glucocorticoid receptor (GR) transcription factors are required for MT expression in response to immune response mediation by IL-6. The SNP-dependent decrease of *MT2A* expression was also reported in a Japanese study [72] in which 17.6% of 119 individuals had an A → G SNP (A/G: 16.8%, G/G: 0.8%) in the *MT2A* promoter near the TATA box (rs28366003). A reporter gene assay using HEK293 cells showed that replacement of A by G reduced *MT2A* expression to 30%–70% after zinc and cadmium treatment. In separate studies, Yoshida *et al.* reported increased cadmium levels in the renal cortex of individuals with (group A) and without (group B) MT accumulation [73,74]. Because MT is a cytoprotective factor against cadmium, group B individuals might be expected to be more sensitive to cadmium toxicity than group A. Although the genetic background of these groups was not examined, group B might be expected to have A/G or G/G genotypes. As shown in Table 2, this SNP is also positively associated with lung, prostate and ductal breast cancer [61,62,63,68]. Another possibility for the difference between groups A and B is the involvement of epigenetic regulation of MT expression.

### 4.2. Epigenetic Regulation of MT Expression

MT expression is also regulated through epigenetic mechanisms. Although MT1/2 is ubiquitously expressed, some cell lines do not express MTs (Figure 3). Jacob *et al.* reported that the suppression of *MT1* expression was caused by promoter-specific DNA methylation [75,76]. Moreover, inhibitors of histone deacetylase (HDAC) and DNA methyltransferase (DNMT) synergistically activate its expression [77]. In MT1/2-expressing cells, the HDAC p300 is involved in a zinc-induced MTF-1-containing complex [78]. MTF-1 deletion mutant analysis revealed that this complex plays an essential role in the activation of *MT1* transcription. Furthermore, zinc rapidly and locally disrupts the *MT1* promoter chromatin structure by nucleosome removal. Binding of MTF-1 to the *MT1* promoter was required to initiate histone exclusion, but was not necessary to maintain this exclusion, at least in the short term [79]. *IL-2* transcription has been reported to occur earlier in zinc re-stimulated mouse T cells than at the first stimulation [80], and this is thought to reflect histone exclusion from the *MT1* promoter, which acts as a memory of the first zinc exposure.

The epigenetic mechanisms involved in *MT* transcription are not fully understood, although their disruption modifies *MT* transcription. Chromatin remodeling complexes such as the SWI–SNF complex are not required for cadmium-induced mouse *MT1* transcription [81], while prenatal zinc deficiency affects cadmium-induced mouse *MT2* transcription through epigenetic mechanisms [82]. Hexavalent chromium (Cr^6+^), a heavy metal known for over 100 years to be a human carcinogen, inhibits mouse *MT1* transcription by modifying the transcription potential of p300 [83]. Cross-linking of HDAC1–DNMT1 complexes to chromatin might be involved in the inhibition of *MT* transcription [77,84,85]. These epigenetic mechanisms might influence the biochemical functions of MT.

## 5. Cellular Zinc Homeostasis Involving ZIP and ZnT Zinc Transporters

### 5.1. Zrt- and Irt-Like Proteins (ZIP) and Zn Transporters (ZnT) Transporters

**Figure 4:**
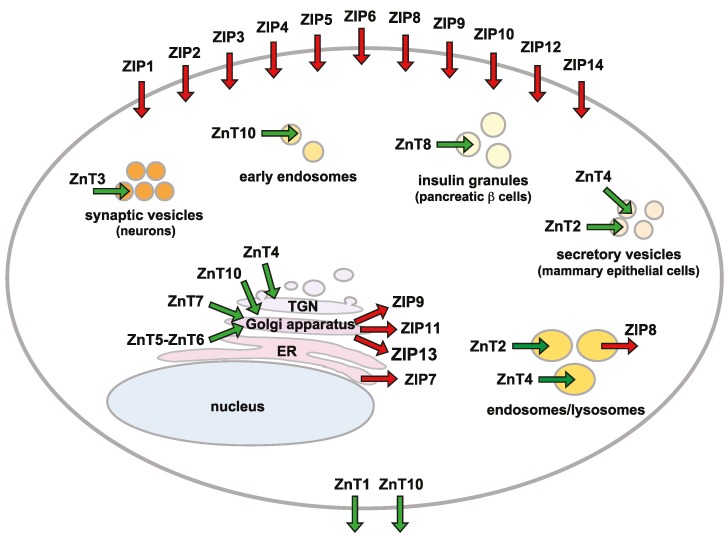
The subcellular localization of ZIP and ZnT transporters. The primary localization of ZIP (**red** arrows) and ZnT (**green** arrows) transporters is shown according to available information. This schematic illustrates a static view of their localization. Cytosolic zinc is mobilized into or out of different subcellular compartments, including synaptic vesicles or insulin granules in a cell-specific manner. ER, endoplasmic reticulum; TGN, *trans*-Golgi network.

In addition to the chelating and releasing by MTs, mobilization of zinc across biological membranes is important to maintain cellular and subcellular zinc homeostasis. Although zinc ions can cross biological membranes through various calcium channels, ZIP and ZnT transporter family proteins play crucial roles as transport routes [86,87] (Figure 4). ZIP transporters mobilize zinc from the extracellular space or intracellular organelles to the cytosol, while ZnT transporters function in zinc efflux and compartmentalization as cation diffusion proteins.

A total of 14 ZIP and nine ZnT transporter genes are encoded in mammalian genomes. Each of the zinc transporters shows a tissue-specific, developmental, stimulus-responsive expression pattern, and specific cellular and subcellular localization. Both transporters display specific changes in protein stability and cellular localization in response to various stimuli including zinc deficiency or excess [2,10,11,12]. Recent studies have indicated that epigenetic expression control occurs in a number of ZIP and ZnT transporters, as seen for *MT* genes (Section 4.1) [88,89]. Moreover, micro RNA-mediated expression control has also been revealed to control cellular zinc homeostasis [90,91]. Molecular information about both zinc transporters, including expression regulation at the protein level, discrimination of substrate metal, and transport mechanism, is extensively reviewed elsewhere [2,12,92], so is not covered here.

### 5.2. Overview of ZIP Transporter Knockout Animals and Human Diseases

*Zip1*, *Zip2*, and *Zip3* KO mice are more likely to produce abnormal embryos when dietary zinc is deficient [93,94,95,96]. *Zip3* KO mice also show zinc retention in the secreted milk pool [97], while *Zip1* and *Zip3* double KO mice reduce seizure-induced CA1 neurodegeneration [98], suggesting the involvement of ZIP3 in zinc reuptake from milk secreted from mammary glands and the involvement of ZIP1 and ZIP3 in neural degeneration induced by zinc entry. *ZIP4* has been identified as the gene responsible for a rare autosomal-recessive inherited zinc deficiency, acrodermatitis enteropathica (AE) [99,100]. AE is caused by impaired intestinal absorption of zinc, and is characterized by eczematous dermatitis, diarrhea and alopecia [101,102]. The importance of ZIP4 in zinc absorption and intestinal integrity was also confirmed by intestine-specific conditional *Zip4* KO mice [103] because complete *Zip4* KO mice are embryonically unviable [104].

Missense and nonsense mutations of *ZIP5* have been shown to be associated with nonsyndromic high myopia [105], although this has not been investigated using *Zip5* KO mice. However, complete and tissue-specific *Zip5* KO mice were used to show the participation of ZIP5 in the control of zinc excretion [106]. *ZIP8* SNPs (such as rs13107325, resulting in A391T substitution) were associated with the circulation of high-density lipoprotein cholesterol [107] and blood pressure [108]. Hypomorphic *Zip8* mice show utero and neonatal lethality because of multiple organ hypoplasia [109], while chondrocyte-specific conditional *Zip8* KO mice suppress surgically induced osteoarthritis pathogenesis [110], indicating that ZIP8 induces the cartilage breakdown of osteoarthritis [110]. Recently, loss-of-function *ZIP8* mutations have been associated with human diseases including intellectual disability, cerebellar atrophy, severe infantile spasms with hypsarrhythmia, disproportionate dwarfism, developmental delay and hypotonia, strabismus, and cranial asymmetry [111,112].

B-cell-specific conditional *Zip10* KO mice show splenoatrophy with reduced peripheral B cell numbers and diminished immunoglobulin levels, indicating the involvement of ZIP10 in anti-apoptotic signaling in early B-cell survival [26]. Both T-cell-dependent and independent immune responses are attenuated in the mature B cells of *Zip10* KO mice, revealing its importance in the modulation of B-cell receptor signaling [27]. Recent analysis using *ZIP12* KO rats showed that ZIP12 regulates the pulmonary vascular response to chronic hypoxia [113], while *ZIP13* mutations have been shown to cause the spondylocheiro dysplastic form of Ehlers–Danlos syndrome (SCD-EDS) [114,115], which is characterized by hard and connective tissue abnormalities. *Zip13* KO mice show delayed growth, and skeletal and connective tissue abnormalities, which are phenotypes similar to those of SCD-EDS patients [115]. Finally, *Zip14* KO mice exhibit dwarfism, impaired skeletogenesis [116], hypoglycemia, greater body fat, and higher insulin levels than wild-type [117]. Moreover, hepatocyte proliferation is decreased in *Zip14* KO mice during liver regeneration [118]. The study using *Zip14*-KO mice revealed its involvement in the uptake of plasma non-transferrin bound iron by the liver and pancreas, and thus possibly in iron overload in hereditary hemochromatosis [119].

### 5.3. Overview of ZnT Transporter Knockout Animals and Human Diseases

KO mice of ZnT transporters also indicate their crucial roles in zinc-related pathophysiology. *Znt1* KO mice are embryonically unviable from an early stage because of the impaired zinc transfer from the mother [120]. *ZnT2* has been identified as the gene responsible for transient neonatal zinc deficiency (TNZD), which is caused by low zinc levels in breast milk [121,122,123,124,125]. The symptoms are similar to AE, but TNZD only develops in breast-fed infants, and does not reoccur after weaning. *ZnT2* KO mice showed the importance of ZnT2 during lactation and mammary gland development [126], while *ZnT3* KO mice show age-dependent defects in learning and memory such as spatial working and fear [127,128,129]. They also lack synaptic zinc [130] and display differences in protein and gene expression important in neurotransmission [127], suggesting modulation functions of ZnT3 in synaptic transmission and plasticity. Moreover, loss of ZnT3 function increases the risk of febrile seizures in humans [131].

A spontaneous *Znt4* mutant mouse produces milk with reduced zinc levels, so-called lethal milk because pups nursed by these dams die before weaning [132]. Recently, the *Znt4* mutant mouse was shown to have defects in mammary gland secretion and hallmarks of precocious involution during lactation [133]*. Znt5* KO mice display poor growth, osteopenia, and male-specific sudden cardiac death [134], and also show cytokine production defects in mast cells, which is mediated by the high-affinity immunoglobulin E receptor [135]. *Znt7* KO mice show poor growth, decreased adiposity, and mild zinc deficiency, while males also have high-fat diet induced-insulin resistance and glucose intolerance [136]. Nonsynonymous *ZnT8* SNPs (rs13266634, resulting in R325W substitution) are known to increase the risk of type 2 diabetes [137,138,139,140], which is attributed to the lower zinc transport activity of the risk allele although the precise molecular mechanism of this requires further investigation because *Znt8* KO mice phenotypes have been variable in sex and genetic background [2,141]. Nevertheless, all *Znt8* KO mice have defects in the formation of zinc–insulin crystals [142,143,144,145,146]. Contrary to these findings, a recent finding showed that the single nucleotide variants causing the truncation of ZnT8 protect against type 2 diabetes in heterozygous individuals [147]. This discrepancy also needs further investigation from the viewpoint of ZnT8 zinc transport activity. The rs13266634 SNP is also a determinant of humoral autoreactivity to ZnT8 [148]. Finally, homozygous *ZnT10* mutations are involved in Parkinsonism, which is characterized by hypermanganesemia, hepatic cirrhosis, polycythemia, and dystonia [149,150]. Recent molecular analysis indicates that ZnT10 is functional in the detoxification of cellular manganese [151].

We briefly summarize information about ZIP and ZnT transporter mutations in human genetic diseases in Table 3. Moreover, many SNPs have been reported in ZIP and ZnT transporter genes that are suggested to be associated with human diseases. This information is summarized elsewhere [2,152].

## 6. Cooperative Functions of MT and ZnT Transporters in Cellular Events

### 6.1. Cooperative Regulation of MT and ZnT Transporters Controls Cytosolic Zinc Homeostasis

The maintenance mechanisms of cellular zinc homeostasis are known as “zinc buffering” and “zinc muffling” [60,155]. This buffering mechanism is important to maintain a zinc ion concentration in the cytosol in the pM range and is achieved by cytosolic zinc-binding proteins including MTs. The muffling mechanism is functional under non-steady conditions, in which transient changes in zinc ion concentrations in the cytosol are modulated by zinc-binding proteins such as MTs and zinc transporters through moving zinc ions into subcellular compartments or out of cells [60,155]. When free zinc ion concentrations in the cytosol are sufficiently high, MTF-1 induces the transcription of *MT-1* and *-2* and several ZnT transporters, and their cooperative expression contributes to the maintenance of cellular zinc homeostasis [32,34,35,36]. Thus, the cooperative regulation of MT and ZnT transporters is essential to control cellular zinc homeostasis over a variety of zinc levels.

### 6.2. Cooperative Regulation of MT and ZnT Transporters for the Activation of Zinc-Dependent Ectoenzymes

**Figure 5:**
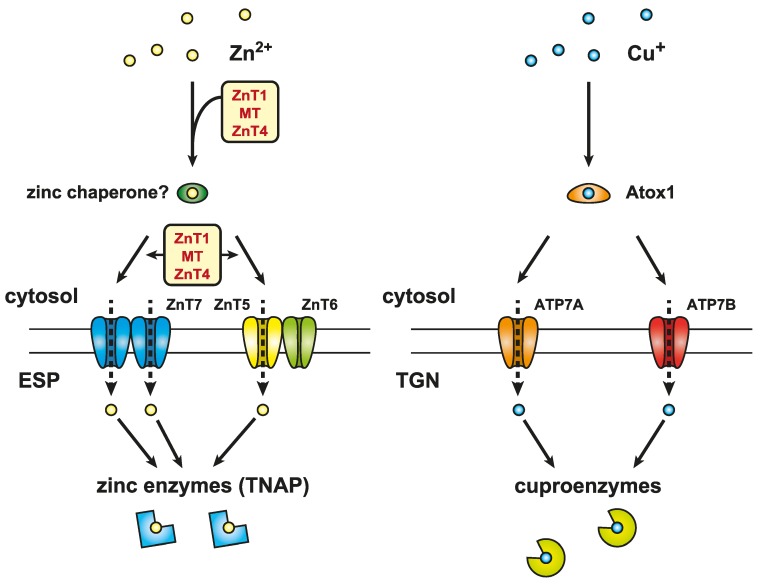
Cooperative function of MT, ZnT1, and ZnT4 in the activation of zinc-requiring ectoenzymes. The facilitated transfer of cytosolic zinc to ZnT5–ZnT6 heterodimers and ZnT7 homodimers may function under cooperative control of ZnT1, MT, and ZnT4 (**left**). *ZnT1MTZnT4* KO cells exhibit significantly reduced TNAP activity (**left**), which is reminiscent of the phenotypes of cytosolic copper chaperone *Atox1*-deficient cells (**right**). Atox1 plays a crucial role as a copper chaperone in transferring cytosolic copper to two copper-transporting P-type ATPases, ATP7A and ATP7B, located in the *trans*-Golgi network (TGN). This therefore contributes to the activation of copper-requiring ectoenzymes (cuproenzymes). Considering the high level of analogy between *ZnT1MTZnT4* KO and *Atox1*-deficient cells, a putative zinc chaperone under the cooperative control of ZnT1, MT, and ZnT4 is hypothesized to play a crucial role in facilitating the transfer of cytosolic zinc to ZnT5–ZnT6 heterodimers and ZnT7 homodimers (not shown) located in the early secretory pathway (ESP). This then contributes to the proper activation of zinc-requiring ectoenzymes such as TNAP (**left**).

In addition to the maintenance of cellular zinc homeostasis, the cooperative functions of MTs and zinc transporters likely contribute to various biological events. However, the molecular evidence for this is limited. We recently studied the activation process of a zinc-requiring ectoenzyme, tissue non-specific alkaline phosphatase (TNAP), to more thoroughly investigate this [156]. The activation of TNAP needs ZnT5–ZnT6 heterodimers and ZnT7 homodimers of the early secretory pathway as a zinc entry route into the lumen of the pathway [157,158,159]. Unexpectedly, cells lacking MT, ZnT1, and ZnT4 (*ZnT1^−/−^MT^−/−^ZnT4^−/−^*) also show significantly reduced TNAP activity, in spite of normal operation of ZnT5–ZnT6 heterodimers and ZnT7 homodimers and increased cytosolic zinc levels [123,156]. Interestingly, the impairment of TNAP activation in *ZnT1^−/−^MT^−/−^ZnT4^−/−^* cells is reversed by excess zinc supplementation [156], strongly suggesting that the transfer of cytosolic zinc to ZnT5–ZnT6 heterodimers and ZnT7 homodimers may be facilitated under the cooperative control of ZnT1, MT, and ZnT4 (Figure 5).

The phenotypes of *ZnT1^−/−^MT^−/−^ZnT4^−/^*^−^ cells are somewhat similar to those of cytosolic copper chaperone *Atox1*-deficient cells, in which intracellular copper levels are increased [160,161] but the activity of secretory cuproenzymes is significantly reduced [162,163]. However, the impairment of cuproenzyme activation is recovered by excess copper supplementation to the cells [162,163]. Atox1 plays a crucial role as a copper chaperone in the facilitated transfer of cytosolic copper to copper-transporting ATPases (ATP7A and ATP7B) to activate secretory cuproenzymes in the *trans*-Golgi network [55,56,164]. Considering the phenotypic analogies between *ZnT1^−/−^MT^−/−^ZnT4^−/−^* cells and *Atox1*-deficient cells, it is interesting to hypothesize the presence of cytosolic zinc chaperone proteins, which conduct the facilitated transfer of cytosolic zinc to ZnT5–ZnT6 heterodimers and ZnT7 homodimers. Both ZnT1 and ZnT4 are membrane proteins, while MT is a cytosolic protein. Thus, MT may be functional as a cytosolic chaperone through its cooperative operation with ZnT1 and ZnT4, although there is no experimental evidence as yet for this. This type of zinc chaperone protein has been speculated based on the molecular modeling of bacterial ZnT homologs, which may have an Atox1-like structure, and appears to dock to the intracellular cavity between transmembrane domains and carboxyl-terminal cytoplasmic domains of ZnT transporters [165].

## 7. Perspectives

The field of zinc metabolism and homeostasis has undergone a dramatic expansion indicating in recent decades, which has revealed that zinc plays important roles in a variety of biological processes. However, the molecular basis underlying these mechanisms has only relatively recently been identified. Many questions remain to be answered with respect to zinc metabolism and its involvement in the divergent array of physiological and pathophysiological processes. These include how zinc ions are sensed and recognized by proteins including MTF-1, MT, and ZIP and ZnT transporters. Moreover, how do MT and ZIP and ZnT transporters operate correctly during zinc transfer to target proteins/enzymes through zinc release via ligand-centered reactions in zinc-thiolate coordination in MT, or zinc mobilization across the biological membranes by ZIP and ZnT transporters? How are these processes activated and controlled in terms of both timing and location? In connection with this, is a zinc chaperone operative in cellular zinc metabolism? Furthermore, how are MT, ZIP and ZnT transporter gene expression controlled epigenetically? Finally, why are so many MTs present in humans and do they have an isoform-specific function? Answers to these questions should provide an important direction for future work on zinc as well as an understanding of the roles of zinc in health and disease.

## Figures and Tables

**Table 1:** Knockout (KO) phenotypes of *MT1/2*.

Phenotype	Strain	References
Metal binding		
Increased sensitivity to heavy metal toxicity	129/Sv, C57BL/6	[16,17,19]
Increased sensitivity to zinc deficiency and excess	C57BL/6	[18]
Decreased FcεRI-induced IL-4 production, which is mediated by calcineurin (CaN)/nuclear factor of activated T-cell (NFAT) signaling, in basophil granulocytes	C57BL/6	[25]
Reduced survival in Cu/Zn-superoxide dismutase (SOD1)-mutated (G93A) mice, which is a familial mouse model of amyotrophic lateral sclerosis (ALS)	129/Sv	[30]
Reactive oxygen species (ROS) scavenging		
Increased sensitivity to X-irradiation-induced bone marrow injury	C57BL/6	[20]
Increased chemical and radiation-induced carcinogenesis	C57BL/6	[21]
Increased *Helicobacter pylori (H. pylori)*-induced gastric erosive lesions with infiltration of leukocytes	C57BL/6	[24]
Unknown mechanisms		
Increased sensitivity to lipopolysaccharide (LPS)/d-galactosamine-induced lethality	129/Sv	[22]
Increased coagulatory and fibrinolytic disturbance and multiple organ damage induced by LPS	C57BL/6	[23]
High-fat-diet-induced obesity, increased plasma leptin and leptin mRNA in the white adipose tissue when fed the high-fat-die (a leptin-resistant state)	129/Sv	[28]
Shorten the lifespan, exhibiting signs of weight loss, hunchbacked spines, lackluster fur and an absence of vigor in male living beyond the mean lifespan	129/Sv	[29]

**Table 2:** Specific MT1/2 isoform functions.

Isoform	Isoform Specific Function	Findings	Ref.
MT1A	Increase risk of lung cancer	Single nucleotide polymorphisms (SNPs) (rs7196890)	[68]
MT1A, 1G	Regulate myeloid differentiation	Negatively regulated by PU.1 in leukemia cells (in microarray analysis) Inhibition of retinoic acid-induced differentiation by *MT1G* overexpression.	[69,70]
MT1X	Mediate cisplatin-induced apoptosis	Interacts with Akt and tongue cancer resistance-associated protein 1 (TCRP1) in oral squamous cell carcinoma (in microarray analysis)	[67]
		Increased sensitivity to cisplatin through activation of phosphatidylinositol-3-kinase (PI3K)/Akt/nuclear factor-κB (NF-κB) signaling pathway by knockdown of MT1X with TCRP1	
MT2A	Regulate autophagy and apoptosis	Inhibition of intracellular free zinc elevation by knockdown of MT2A	[66]
		Interacts with homeobox containing 1 (HMBOX1) (in yeast two-hybrid assay), overexpression of which increases intracellular free zinc	
		Inhibition of anti-apoptosis and pro-autophagy effects of HMBOX1 by zinc chelator, *N*,*N*,*N'*,*N'*-Tetrakis(2-pyridylmethyl)ethylenediamine (TPEN)	
MT2A	Increase risk of prostate cancer	SNPs (rs28366003)	[61,62]
MT2A	Increase risk of ductal breast cancer	SNPs (rs28366003)	[63]

**Table 3:** Zinc transporter mutations reported to be involved in inherited diseases.

Gene	Disease	MIM No.	Clinical Features	Pattern of Inheritance	References
*SLC39A4/ZIP4*	Acrodermatitis enteropathica (AE)	201100	Eczematous dermatitis on the perioral, perianal, and areas, alopecia, diarrhea, growth retardation because of decreased zinc absorption, Ameliorated with zinc supplementation.	Homozygous, Compound heterozygous, Dominant negative	[99,100,101,153,154]
*SLC39A5/ZIP5*	Nonsymptomatic high myopia	615946	Refractive error, tigroid and focal atrophy of choroid.	Heterozygous	[105]
*SLC39A8/ZIP8*	Cerebellar Atrophy Syndrome, a type II congenital disorder of glycosylation (CDG)	-	Intellectual disability, cerebellar atrophy, cranial asymmetry, dysproportionate dwarfism, severe infantile spasms with hypsarrhythmia, hypotonia, strabismus.	Homozygous, Compound heterozygous	[111,112]
*SLC39A13/ZIP13*	spondylocheiro dysplastic Ehlers-Danlos syndrome (SCD-EDS)	612350	Postnatal growth retardation, skeletal and connective tissue abnormalities, finger contractures, joint hypermobility, protruding eyes with bluish sclera, decreased hydroxyl collagen levels.	Homozygous	[114,115]
*SLC30A2/ZnT2*	Transient neonatal zinc deficiency (TNZD)	608118	Erosive dermatitis around the mouth, genital region, neck, and fingers, diarrhea, hair loss, alopecia, Ameliorated with zinc supplementation to infants.	Dominant negative, Heterozygous, Compound heterozygous	[121,122,123,124,125]
*SLC30A3/ZnT3*	Increased risk of febrile seizures	-	Potentially a prelude to more severe epilepsy.	Heterozygous	[131]
*SLC30A10/ZnT10*	Hypermanganesemia, syndrome of hepatic cirrhosis, dystonia, polycythemia	613280	Dysarthria, hypertonia, fine tremor, bradykinesia, spastic paraparesis, Improved by metal chelation therapy.	Homozygous	[149,150]

## Abbreviations

AE	acrodermatitis enteropathica
ALS	amyotrophic lateral sclerosis
CDG	congenital disorder of glycosylation
CaN	calcineurin
DNMT	DNA methyltransferase
ER	endoplasmic reticulum
ESP	early secretory pathway
GR	glucocorticoid receptor
HDAC	histone deacetylase
HMBOX1	homeobox-containing 1
IL	interleukin
KO	knockout
LPS	lipopolysaccharide
MT	metallothionein
MBD	methyl CpG binding protein
MTF-1	metal response element-binding transcription factor-1
NFAT	nuclear factor of activated T-cell
NF-κB	nuclear factor-κB
PI3K	phosphatidylinositol-3-kinase
SCD-EDS	spondylocheiro dysplastic form of Ehlers–Danlos syndrome
SLC	solute carrier
SNPs	polymorphisms
SOD	superoxide dismutase
STAT3	signal transducer and activator of transcription
TCRP1	tongue cancer resistance-associated protein 1
TGN	*trans*-Golgi network
TNAP	tissue non-specific alkaline phosphatase
TNZD	transient neonatal zinc deficiency
TPEN	*N*,*N*,*N'*,*N'*-Tetrakis(2-pyridylmethyl)ethylenediamine
ZIP	Zrt- and Irt-like protein
ZnT	Zn transporter
ZNF	zinc finger transcription factor
